# Counting the CHIPs: The High-Stakes Role of Clonal Hematopoiesis in Multiple Myeloma

**DOI:** 10.3390/biomedicines14040899

**Published:** 2026-04-15

**Authors:** Morgan Szalay, Ian Seguel Strange, Kyle Banwell, Sophia Campana, Adina Dass, Sereen Hej-Ali, Halima Mohamed, Sahar Khan

**Affiliations:** 1 Department of Biomedical Science, Faculty of Science, University of Windsor, Windsor, ON N9B 3P4, Canada; 2 School of Medicine, Toronto Metropolitan University, Toronto, ON L6T 2T9, Canada; 3 Windsor Regional Hospital, Windsor, ON N8W 1L9, Canada; 4 Department of Oncology, Schulich School of Medicine and Dentistry, Western University, London, ON N6A 3K7, Canada

**Keywords:** clonal hematopoiesis, multiple myeloma, ASCT, BCMA, IMiDs

## Abstract

Clonal hematopoiesis of indeterminate potential (CHIP) is the clonal expansion of somatically mutated hematopoietic stem cells (HSCs) in the bone marrow. CHIP mutations are relatively common in multiple myeloma (MM) and have been identified as potential biomarkers for poorer survival outcomes. MM is a hematological malignancy that, despite treatment advances, remains aggressive and incurable for many patients. The potential impact of CHIP mutations on the outcomes of MM treatments has been the topic of several recent studies, yet both the magnitude and the modality by which CHIP exerts its negative effects on treatment and disease progression remain to be fully elucidated. Evidence suggests that CHIP mutations may contribute to inferior survival and treatment tolerances, as well as contribute to greater treatment toxicity and related frailty. In this review, we synthesize and discuss the available literature to provide an updated understanding of the complex role that CHIP plays in altering the MM microenvironment, and the resulting impact on standard MM treatments, autologous stem cell transplant (ASCT) and B-cell maturation antigen (BCMA)-targeted therapy/CAR-T, and the important role of immunomodulatory drug (IMiD) maintenance therapy in clinical outcomes.

## 1. Introduction

Clonal hematopoiesis of indeterminate potential (CHIP) is defined as clonal expansion of hematopoietic cells due to acquired somatic mutations [1,2]. In particular, these mutations occur within myeloid malignancy-associated genes in the blood or bone marrow, at a commonly accepted variant allele frequency (VAF) threshold of 1–2% [2,3]. CHIP mutation prevalence increases with age, commonly being observed in elderly individuals with no prior history of hematological malignancies [1,2,4]. CHIP has been identified as a potential factor influencing clinical outcomes of patients with non-myeloid hematological malignancies, including multiple myeloma (MM) [5]. The current literature suggests that CHIP may contribute to MM progression in a gene-specific manner through clonal expansion and alterations to the MM microenvironment. However, the precise biological mechanisms and the extent by which CHIP does so remains to be fully elucidated.

CHIP’s role in MM has been the focus of several recent studies (summarized in Table 1) investigating its potential as a promising, partially validated biomarker. CHIP has been identified in MM patients relatively frequently, with some reporting variation due to differences between cohorts including demographics like age, disease stages, underlying disease characteristics, and the treatments received. Differences in sample type, sequencing methods, and VAF thresholds are also contributors to variability in reported incidence. In a study by Mouhieddine et al. (2020) with a sample size of 629 MM patients prior to autologous stem cell transplant (ASCT), 21.6% had at least one CHIP mutation at a VAF ≥ 1.0% [5]. Alternatively, in a study including 101 pre-ASCT patients with MM, CHIP prevalence was 23% at a VAF threshold of ≥2.0% [6]. Of the 10 studies summarized in Table 1, the reported incidence of CHIP in MM ranged from 10% to 55%, at varying disease and treatment stages. Collectively, these findings indicate that CHIP is relatively common in MM and raise important questions regarding its potential clinical significance.

### 1.1. Clinical Associations of CHIP in MM Patients

Studies demonstrate that certain features of clonal hematopoiesis (CH), including high-risk mutations, multiple mutated genes, or higher VAFs, correlate with poorer overall survival (OS) and progression-free survival (PFS) in some cohorts [5,15]. A retrospective multi-center analysis indicated that MM patients harboring CH experienced significantly shorter OS and PFS compared to those without CH [14]. Additionally, preliminary data from a 317 MM patient cohort reported diminished OS among individuals with CHIP (8.6 vs. 14.5 years) and suggested a potential link to poor survival [16]. However, these findings have not been consistently replicated in larger cohorts. For instance, a later cohort study done by Mouhieddine et al. (2023) identified CH in 10% of 986 newly diagnosed MM patients [7]. Notably, the presence of CH at initial diagnosis showed no correlation with variations in either OS or PFS [7]. These conflicting results illustrate how CHIP remains a partially validated biomarker rather than an established determinant of survival due to inconsistency in clinical impact across different patient populations and treatment contexts.

Aside from their role in hematological malignancies, CHIP mutations in the general population are increasingly associated with a spectrum of non-malignant conditions, most robustly in cardiovascular disease (CVD). In 2014, studies identified CHIP as a contributor to an increased risk of coronary heart disease (HR 2.0), ischemic stroke (HR 2.6), and all-cause mortality (HR 1.4) [3]. Further research in 2017 reinforced these findings, demonstrating a 1.9-fold increase in coronary heart disease and a 4.0-fold increase in early-onset myocardial infarction (MI) [3,17]. These associations have been linked to mutations in genes such as TET2 and DNMT3A, which predict mortality and hospitalization due to heart failure (HR 2.09). TET2 and ASXL1 clones are also associated with atrial fibrillation and cardiac remodeling [18,19,20,21], while other studies link CHIP to aortic valve stenosis (HR 1.38), plaque vulnerability, ST-Elevation MI (STEMI) outcomes (adjusted HR ~1.9), venous thromboembolism (VTE) (TET-2 associated), ischemic stroke, and post-stroke mortality [22,23,24,25,26,27]. CHIP mutations have also been associated with non-cardiovascular conditions like metabolic syndrome, chronic kidney disease progression, anemia, chronic liver disease, chronic obstructive pulmonary disease, inflammatory bone loss, osteoporosis, and gout [28,29,30,31,32,33,34,35,36,37,38], suggesting a common inflammatory mechanism underlying these associations.

In the context of MM, these conditions are particularly relevant because MM patients already exhibit an increased risk of cardiovascular complications [39]. A 2023 study done by Rhee et al. determined that the presence of CHIP at the time of HCT was associated with a greater risk for developing CVD post-transplant. Among 1036 MM patients who underwent a first ASCT, 19.4% had at least one CHIP-associated mutation at the time of transplant (VAF ≥ 2.0%) [12]. The researchers found that the 5-year incidence of CVD in CHIP-positive ASCT recipients was significantly higher compared to those without CHIP (21.1% vs. 8.4%) [12]. The incidence of individual CVD events, including heart failure, coronary artery disease and stroke, was similarly higher in the CHIP group compared to the non-CHIP group [12]. Gelli et al. (2024) [14] identified an increased prevalence of frailty in MM patients harboring CHIP, with a significant increase in some treatment-emergent adverse effects. This included a higher overall incidence of grade ≥3 toxicities in patients with CHIP versus those without (85.7% vs. 36.5%; *p* = 0.036); higher rates of hematological toxicity including anemia and thrombocytopenia; non-hematological toxicity including peripheral neuropathy, SARS-CoV-2 infections (45.7% vs. 9%; *p*  =  0.012), as well as higher incidence of VTE (51.4% vs. 17%) [14]. This suggests that the presence of CHIP has the potential to operate as a biomarker for poorer treatment outcomes and the presence of co-morbidities in MM.

### 1.2. Mutational Landscape of CHIP in Multiple Myeloma

The mutational landscape of CHIP in MM is complex, with certain genes involved in epigenetic regulation (DNMT3A, TET2, and ASXL1) being among the most commonly mutated, followed by splicing factors (SF3B1, SRSF2, and U2AF1), DNA damage response genes (PPM1D and TP53), and the JAK2 signaling pathway [40]. With some variation (Table 2), these trends of CHIP mutation prevalence are generally conserved across MM cohorts, even with differing sampling methods and detection sensitivity [6,7,13,41]. When characterizing the mutational landscape of CHIP in MM, direct comparison of mutation prevalence across studies is challenging due to variation in cohort size and methodology. For example, studies have employed both next-generation sequencing (NGS) [5,6,7,13,41] and whole-exome sequencing (WES) [7]. There is also variation within individual approaches: although a VAF threshold of 1–2% [5,7,12] is commonly used, this is not always the case, and detection thresholds are not consistently specified [6,41]. Additionally, the composition and size of the gene panels used in targeted NGS vary considerably, ranging from 42 to 66 genes [6,13,41], to large panels of 224 and 568 genes [5,7]. However, despite this methodological variation, a mutational pattern has been demonstrated with epigenetic regulators being the most frequently mutated, followed by splicing factors (Table 2).

Despite mechanistic differences across genes, epigenetic and splicing factor mutations can result in a mutated phenotype, prioritizing hematopoietic stem cell (HSC) proliferation at the cost of cellular differentiation [42,43,44,45,46]. DNA damage response and JAK2 signaling mutations promote clonal survival by diminishing the apoptotic response and activation of the JAK–STAT signaling pathway respectively [47,48,49,50]. Additionally, mutations like TET2, DNMT3A and JAK2 can promote an inflammatory and immunosuppressive bone marrow microenvironment, favoring mutant HSC expansion, sustaining clonal dominance, driving CHIP-related disease progression, and modulating the MM microenvironment [51,52,53,54,55,56]. Mutations in less frequently altered genes such as SF3B1, NF1, KRAS, and NRAS have also been identified in CHIP, although their contributions to clonal expansion and disease progression remain less clearly defined [40]. For example, mutant SF3B1 has been linked to the ubiquitination of cell cycle regulators implicated in oncogenesis [52], and among MM patients exhibiting myelodysplastic (MDS)-like phenotypic changes, half had CH detected at diagnosis, frequently involving TET2 and NRAS mutations [57]. In this context, CHIP not only coexists with MM, but may also contribute to alterations in the bone marrow tumor microenvironment and influence disease progression in a mutation-specific manner.

A study including 106 patients with MM, done by Lionetti et al. (2025), showed that CHIP can further modulate the MM microenvironment through proinflammatory and immunosuppressive effects [11]. Using single-cell RNA sequencing and a differential expression analysis in 16 patients with MM, it was shown that CHIP-positive cells had some differences [11]. CHIP-positive monocytes exhibit enhanced M1 polarization while both CHIP-positive monocytes and T cells show elevated production or signaling of proinflammatory cytokines like IL-6, similar to what is seen in those patients with advanced-stage MM [11]. MM itself is also known to drive the evolution of CH through extracellular vesicles released by neoplastic plasma cells, which dysregulate HSC and progenitor cell differentiation through BM inflammation, significant enough to trigger the clonal expansion required for CH [57]. One study reported an increasing prevalence of CH from 5.8% prior to treatment to 25% afterwards [57]. Mouhieddine et al. (2020) also found that in 8 of 10 patients with therapy-related myeloid neoplasms (TMN), the driver mutation present prior to ASCT had expanded by TMN diagnosis [5]. The longitudinal data on clone dynamics is divided as the majority of patients demonstrated stability, with 21 of the 27 CHIP-negative patients remaining that way throughout treatment, and even some of the positive cases maintaining comparable allele frequencies for specific mutations. In contrast, other patients displayed clonal expansion through linear or branching evolution, as seen in new TP53 mutations or significant changes in the VAF of existing clones during therapy [11]. Together, these findings support a bidirectional relationship in which CHIP reshapes the BM microenvironment while the MM-driven inflammatory environment reciprocally fosters CH, reinforcing disease progression [57].

## 2. Impact of CHIP on Therapeutic Outcomes in MM

### 2.1. Impact of CHIP on ASCT Outcomes

The use of ASCT is the standard of care for most eligible patients with MM [58]. However, high-dose chemotherapy paired with the mobilization of HSCs is associated with both short- and long-term treatment-related toxicity [59]. The role of CHIP in the toxicity and outcomes of ASCT in patients with MM has been the focus of several recent studies. CHIP has been identified frequently in MM patients undergoing ASCT and has been associated with a negative impact on ASCT recovery, but these findings remain inconsistent [5,6,13].

Some emerging evidence suggests that CHIP may negatively influence the OS of MM patients undergoing ASCT. In the previously mentioned study by Mouhieddine et al., the researchers identified an association between CHIP and both decreased OS and PFS but did not associate these findings with increased risk of CVD or therapy-related myeloid neoplasm (t-MN) [5]. A median OS of 5.3 years for the patients with CHIP was reported, significantly lower than those without, at a median OS of 7.5 years [5]. The leading cause of decreased survival was attributed to increased myeloma progression. Notably, a later and larger study done by Mouhieddine et al. (2023) included 986 patients with MM, both transplant-eligible and -ineligible and found no significant difference in OS and PFS between the patients with CHIP compared to those without [7]. The researchers attributed this variation largely to differences in treatment exposure between the cohorts, particularly the use of immunomodulatory drug maintenance therapy (IMiDs), which has inconsistently been shown to mitigate the impact of CHIP on OS and PFS in ASCT-receiving MM patients [5,6].

Other recent studies have also reported lower OS and PFS in MM patients post-ASCT. In a study done by Gelli et al., CHIP was associated with an increase in biomarkers associated with disease aggressiveness including b2microglobulin, serum free light chains, urinary protein excretion, creatinine and estimated glomerular filtration rate. Consistent with this, mosaic plot analysis showed higher prevalence of CHIP mutations amongst higher risk MM patients, as defined by ISS and revised ISS-staging. CHIP was associated with adverse outcomes following ASCT, including shorter PFS and OS (mPFS of 493 days in those with CH vs not reached at 5000 days in control; *p* < 0.0001) and OS (mOS of 925 days in CH carriers vs. not reached at 5000 days in non-carriers) [14]. CHIP carriers were found to have higher levels of disease aggressiveness markers compared to non-CHIP patients.

HSC mobilization and recovery are essential for the efficacy of ASCT. A 2024 study done by Stelmach et al. found that CHIP, specifically DNMT3A and PPM1D mutations, may negatively impact these processes [13]. Stelmach et al. found that MM patients with DNMT3A and PPM1D CH mutations had significantly lower CD34^+^ stem cell yield pre-transplant when compared to the non-CHIP group (median 4.65 × 10^6^ vs. 7.50 × 10^6^ CD34^+^ stem cells harvested) [13]. The researchers also observed significantly lower blood platelet counts pre-ASCT in the CHIP group compared to the non-CHIP group, suggesting that hematopoiesis and HSC function in patients with these mutations are impaired [13]. From the same study, the researchers found that the DNMT3A and PPM1D mutations were associated with a delay in regeneration of blood platelets post-ASCT, highlighting the potential for CHIP-related complications in ASCT recovery. Similarly, Mouhieddine et al. (2020) also found decreased stem cell mobilization efficiency in CHIP-positive patients, reporting 5.8 × 10^6^ CD34+ cells/kg/day, compared to 8.3 × 10^6^ CD34+ cells/kg/day for those without CHIP [5]. This presents a concern as lower mobilization makes transplant more difficult in these CHIP-positive patients.

Multiple studies have shown the increased risk of post-transplant infections that CHIP-positive patients face. From Mouhieddine et al.’s 2023 study, it was found that patients with CH experienced an increased risk of recurrent bacterial infections [7]. Another study found that patients with specific CHIP mutations demonstrated a predisposition to developing infections in general [60]. The literature appears to agree about CHIP mutations increasing the risk of post-transplant infections, which could be due to an impairment of the body’s ability to defend against pathogens. In the previously mentioned study by Lionetti et al. (2025), they found impaired antigen-presenting function in myeloid dendritic cells, which is needed to initiate an immune response against new threats [11]. Another issue many CHIP-positive patients face is an increased rate of hospitalization post-transplant. One larger clonal tracing study of 81 patients post-ASCT, most of whom were being treated for MM, found that patients with CH experienced longer hospitalizations for their transplant recovery and more prolonged neutropenia [10].

### 2.2. The Role of IMiDs

While little research has been done specifically on the interaction between CHIP mutations and IMiDs, some associations have been identified within the literature. In the post-ASCT setting, IMiD maintenance therapy has been identified to reduce the deleterious impact of CHIP on patient PFS and OS to the point that patient outcomes were statistically indistinguishable to those without CHIP [5,13,61]. However, further research is required to determine the validity of these associations, as additional studies have found no significant association between CHIP status and PFS/OS in both transplant and non-transplant cohorts [6,7]. Note that these studies are not directly comparable, with differences in follow-up time, standards of treatment, and methodology of CHIP detection. Alongside the known risks of t-MN and second primary malignancies (SPMs) in MM patients receiving IMiD therapy [5,62,63,64], the presence of CHIP mutations may present an additional risk since it has been demonstrated that TP53-mutated t-MNs are particularly enriched in MM patients treated with thalidomide analogs, especially lenalidomide, through the selective outgrowth of Trp53-mutant HSPCs [65]. Despite this, the current literature suggests no increased risk of t-MN or SPMs among patients with CH [5,6]. Moreover, the consensus is that the detection CHIP mutations should not exclude patients with MM from receiving lenalidomide or other IMiD therapies given their reliably beneficial outcomes [5,7,61,66,67,68,69,70].

### 2.3. Effects of CHIP on Treatment Efficacy and Survival in CAR-T/BCMA

BCMA is a key therapeutic target in MM because it is highly expressed on malignant plasma cells [71]. CAR-T therapies engineered to target BCMA have shown strong clinical activity, with early studies reporting overall response rates of 73–100% and complete response rates of 31–69% in relapsed or refractory disease [72,73,74]. The impact of CHIP mutation was evaluated in a study of 104 patients with MM treated with BCMA-directed CAR-T therapy [8]. Among this cohort, 55% of patients carried CHIP mutations detected prior to CAR-T therapy. The study found no significant differences in OS or treatment response between groups. Median progression-free survival was similar between groups (12.0 months in CHIP patients vs. 11.8 months in non-CHIP patients), and overall survival was 24.6 months in the CHIP group compared with 38.1 months in the non-CHIP group, although this difference was not statistically significant [8]. Response rates were also comparable, with overall response rates of 86% in patients with CHIP and 79% in those without CHIP, and complete response rates of 49% and 38%, respectively [8].

Despite lack of CHIP-associated differences in PFS or OS, increased cytokine release syndrome (CRS) is often reported [75,76]. A genetic analysis of blood specimens from 154 patients with MM or non-Hodgkin lymphoma (NHL) observed higher rates of complete responses and severe CRS in patients with CHIP [75]. A study of 55 patients with MM also found no significant difference in overall response rates (94% with CHIP vs. 91% without), also reported higher CRS incidence and grade in CHIP carriers [77]. Overall, while the impact of CHIP on BCMA-targeting therapies on therapeutic efficacy is likely not significant, increased inflammatory toxicity in CHIP populations is commonly reported. Mature myeloid cells have been proposed as major contributors as CHIP-associated mutations expand their production of immune mediators [75,78]. In vitro suppression of CHIP-related genes in macrophages exemplifies this, leading to a significant increase in proinflammatory cytokines characteristic of CRS [79].

In parallel, recent work has investigated whether CH may influence post-treatment outcomes separate from disease control. In one cohort of 213 CAR-T recipients, NGS data was available for 90 patients prior to therapy, of whom 39% carried CH-associated mutations with VAF ≥ 2.0% [80]. Patients with CHIP were significantly more likely to experience delayed hematologic recovery following BCMA CAR-T therapy, including delayed platelet recovery beyond 100 days (38% vs. 11%, *p* = 0.006) and delayed myeloid recovery (41% vs. 19%, *p* = 0.04), which has been shown to worsen survival outcomes. These findings suggest that underlying clonal hematopoiesis may contribute to prolonged cytopenias and impaired bone marrow recovery after CAR-T treatment, which may negatively influence long-term clinical outcomes in heavily pretreated MM populations [80]. Overall, the CAR-T therapy-related literature suggests that CHIP does not appear to affect survival, except in cases where secondary malignancies develop. CHIP was further associated with toxicities that complicate recovery, yet its potential presents as a prognostic biomarker for predicting the severity of CRS following treatment.

## 3. CHIP and Clonal Evolution

Clonal evolution of CHIP in MM is influenced by selective pressures rather than the simple linear expansion of clones. Factors including chronic inflammation, cytokine signaling, therapeutic exposures, lifestyle behaviors, and aging may favor the outgrowth of high-fitness CHIP mutants over time [40,81,82]. However, clonal size alone is not a reliable predictor of long-term dominance, as clonal architecture remains dynamic throughout disease progression and treatment [81]. The effects of two different maintenance therapies were studied in 94 MM patients who received frontline induction and ASCT. These were maintenance therapy with lenalidomide/Revlimid (R) alone (n = 32) or Carfilzomib, Revlimid and Dexamethasone (KRD) (n = 62); of these, 52 patients discontinued maintenance after achieving minimal residual disease negativity after at least 1 year of maintenance therapy. Using a 2% cut-off threshold, CH was present in 6%, 7% and 30% at pre-R maintenance, pre-KRD maintenance and pre-discontinuation of maintenance. In both R and KRD groups, there was significant expansion of small TP53 mutated clones, in contrast to stable allele frequencies in other CH mutated clones such as DNMT3A. Progressive increase in TP53 mutated clone size with increased VAF of >1% was associated with risk of progression to therapy-related neoplasm. Upon treatment discontinuation, most clones stabilized or regressed; however, approximately 18% of patients demonstrated persistent clonal expansion, which in some cases is associated with the development of a second primary hematological malignancy [83]. Similar observations have been reported in other studies, including the expansion of pre-existing CHIP clones and the emergence of new mutations, such as DNMT3A [7]. Overall CHIP clone size in MM often shows minimal change during disease progression and cytotoxic therapies not preferentially expanding DNMT3A mutant clones, suggesting that CHIP dynamics are largely driven by age-related factors and selective expansion of specific fitness-associated mutations rather than broad therapy-induced clonal shifts [14,83].

## 4. Literature Search and Selection

This work is intended as a narrative review to synthesize the current body of literature covering the complexity of CHIP’s potential role as a biomarker in MM treatment. Relevant studies were identified though targeted searches using the database PubMed, where they were filtered based on recency and clinical relevance. Given the narrative approach, systematic filtering criteria were not applied. Studies were selected based on relevance and areas of conflicting data are highlighted and discussed.

## 5. Conclusions and Future Directions

Clonal hematopoiesis remains an active ground of research in multiple myeloma patients, with an emerging body of literature which suggests high incidence in MM patients as well as a potential role in MM survival. This review is limited by its narrative design.

Unlike lymphoma and other non-myeloid hematological malignancy, early data suggested that the primary impact on outcomes was from adverse disease progression, although mutations were identified in hematopoietic progenitor cells and/or blood, rather than plasma cells. This review of the recent literature reveals a potentially more complex impact of CHIP on MM outcomes, suggesting adverse outcomes may also relate to increased cardiovascular mortality, higher incidence of frailty at baseline, and poorer tolerance of therapies with higher incident hematological and non-hematological toxicity, as well as more complications with cellular therapies such as ASCT and BCMA-based therapies. As such, it is important that future studies focus specifically on examining causes of mortality for CHIP-mutated patients to better risk stratify patient populations for various outcomes and therefore better individualize future treatments.

How CHIP mutations evolve over time and impact outcomes of specific therapeutic modalities also require further evaluation, ideally within prospective, longitudinal trials. While better post-ASCT outcomes have been demonstrated with use of IMiD-based therapies as maintenance, the mechanism of this effect is unclear and not clearly reproducible across studies. Moreover, longitudinal data examining allele frequencies for mutations present at diagnosis shows either stability or progression of mutations over time. Limited longitudinal data suggests that new mutations also continue to arise over time, including in patients on IMiD-based therapies; direct comparison of mutation evolution in patients on IMiDs versus similarly treated patients without IMiDs is lacking. Even less data exists on the impact of evolution of CHIP mutations in patients treated with anti-CD38 monoclonal antibodies and proteasome inhibitors.

Overall, CHIP is an active area of research in patients with multiple myeloma and may serve as a useful biomarker for incorporation in large scale, prospective trials to assess for associations with both disease outcomes, co-morbidity, and therapeutic efficacy and tolerance.

## Figures and Tables

**Table 1:** Recent studies reporting CHIP in multiple myeloma.

First Author	Year	Participant Description	Number of Participants	Median Age	Type of Sample	Sequencing Methods	VAF Threshold	No. of MM Patients with CHIP	Percentage of MM Patients with CHIP (%)	Most Common Mutations	Clinical Implication of CHIP
Mouhieddine [5]	2020	MM patients pre-ASCT	629	58	PB and BM	Targeted sequencing	≥1.0%	136	21.6	DNMT3A, TET2, TP53, ASXL1, PPM1D	Inferior OS and PFS with CHIP
Mouhieddine [7]	2023	MM patients	986	63	PB and BM	WES and targeted sequencing	2–35%	99	10	DNMT3A, TET2, ASXL1, PPM1D, TP53	No significant difference in OS and PFS
Wudhikarn [6]	2021	MM patients pre-ASCT	101	56	Pre-ASCT BM	NGS	≥2.0%	23	23	DNMT3A, TET2, TP53, PPM1D, BRAF	CHIP associated with inferior OS and PFS
Gustine [8]	2025	MM patients immediately prior to undergoing BCMA CAR-T	104	-	Whole BM aspirate	Targeted sequencing	≥2.0%	57	55	DNMT3A, ASXL1, TET2, PPM1D, BCOR (Excluded TP53)	Associated CHIP with delayed hematopoietic recovery following BCMA CAR-T therapy
Testa [9]	2022	Patients with PCDs (including MM)	209 (97 with MM)	-	PB or BM	NGS	≥1.0%	16	17	Only included DNMT3A, TET2, ASXL1 (DTA-CH)	DTA-CH correlated with coronary artery disease and congestive heart failure but not OS and PFS
Ortmann [10]	2019	Patients with lymphoid disease undergoing ASCT (including MM)	81 (59 with MM)	57	Deep-frozen grafts or PB (post-ASCT)	Targeted sequencing	≥2.0%	13	22	DNMT3A, PPM1D, TET2, TP53	Increased risk of gain of clone size post-ASCT
Lionetti [11]	2025	MM patients	106	68	PB and BM	NGS		24	22.6	TET2. DNMT3A, ASXL1, SF3B1, PPM1D, NF1	MM microenvironment disrupted in presence of CHIP
Rhee [12]	2024	MM patients post-first HCT	1036	60	Cryopreserved PB stem cells	NGS	≥2.0%	201	19.4	DNMT3A, ALSX1, TET2, TP53	CHIP increased risk of CVD post-ASCT
Stelmach [13]	2023	MM patients pre-ASCT	457	-	Mobilized stem cell product	Targeted sequencing	≥1.0%	152	33.3	DNMT3A, TET2, PPM1D, TP53	Lower CD34+ stem cell counts in MM patients with CHIP
Gelli [14]	2024	MM patients	76	71	PBMCs	NGS	≥1.0%	35	46	DNMT3A, TET2	CHIP associated with increased frailty

*Note.* All studies used a minimum mutation count of ≥1 to define CHIP. PB = peripheral blood, BM = bone marrow, WES = whole-exome sequencing, NGS = next-generation sequencing, HCT = hematopoietic cell transplant, PBMC = peripheral blood mononuclear cell.

**Table 2:** Recently reported CHIP mutations in MM patient cohorts.

Genes		Reported CHIP Mutation Prevalence (%)				
Function	Name	Mouhieddine et al., 2020 [5] N = 629 n CHIP = 136	Wudhikarn et al., 2021 [6] N = 101 n CHIP = 23	Stelmach et al., 2023 [13] N = 457 n CHIP = 152	Mouhieddine et al., 2023 [7] N = 986 n CHIP = 99	Wobst et al., 2026 [41] N = 1011 n CHIP = 173
Epigenetic Regulators	DNMT3A	39	52	43	46	52
	TET2	15	26	28	22	35
	ASXL1	10	9	5	8	NR
Splicing Factors	SF3B1	3	9	4	1	8
	SRSF2	0	0	0	3	NT
	U2AF1	0	0	0	2	NT
DNA Damage	PPM1D	7	13	9	7	NR
	TP53	13	13	5	4	NR
Signaling Molecules	JAK2	3	0	3	1	4

*Note.* Percentages are rounded to the nearest integer; NT = not tested, NR = not reported.

## Data Availability

No new data was generated for this review. All analyzed data is cited within the article.
